# Optimizing Wood–Hemp–Sodium Silicate Composites for Strength, Extrudability, and Cost in Additive Manufacturing Applications

**DOI:** 10.3390/ma19020357

**Published:** 2026-01-16

**Authors:** Nagendra G. Tanikella, Armando G. McDonald, Michael R. Maughan

**Affiliations:** 1 Department of Mechanical Engineering, University of Idaho, Moscow, ID 83844, USA; tani7947@vandals.uidaho.edu; 2 Department of Forest, Rangeland and Fire Sciences, University of Idaho, Moscow, ID 83844, USA; armandm@uidaho.edu

**Keywords:** hemp, hurd, fiber, thermosets, additive manufacturing, wood, value engineering

## Abstract

Utilizing forestry and agricultural byproducts like wood and hemp residues advance sustainable additive manufacturing (AM), while reducing material costs. This study investigated the development and characterization of wood–sodium silicate composites incorporating hemp hurd and hemp fibers for AM applications. Formulations varied by wood fiber type (unsifted, 40 mesh, and pellet), sodium silicate concentration (50–60 wt%), and hemp hurd content (0–15 wt%). Properties evaluated include particle size and bulk density of the constituent materials, rheological behavior, extrusion performance, composite bulk density, and flexural and compressive strengths. Rheology and extrusion were largely influenced by the liquid content. Mixtures with low liquid content (50 wt% sodium silicate) had high motor power and low viscosity. As liquid content increased, motor power decreased, while viscosity increased up to 55 wt% and then decreased at 60 wt%. Mechanical properties correlated with particle size, where finer particles enhanced strength. A cost analysis was conducted using raw material prices to determine the economic feasibility of each formulation. Finally, the formulations were evaluated based on strength-to-cost ratios, extrudability and processability. The formulation with pellet wood fibers, 55 wt% sodium silicate, and 10 wt% hemp hurd achieved a high ratio of 73.0 MPa/$ while maintaining low motor power. This formulation offered additional benefits which are discussed qualitatively.

## 1. Introduction

Large amounts of wood residue are produced by forest-based industries such as lumber mills during the processing of logs to primary lumber. Globally, an estimated 251 million m^3^ of wood residues were produced in 2023 [1]. These residues have applications in non-structural panels like particleboard and medium density fiberboard, as well as in energy/heat production through char and pellets [2,3,4,5]. The residues, in the form of wood fiber and pellets, are also used in additive manufacturing (AM) applications, both as reinforcing materials and as primary constituents [6,7,8,9,10,11,12,13,14].

The utilization of agro-forestry byproducts, such as wood residues and hemp derivatives, in construction and AM presents numerous advantages over traditional materials. Environmentally, these byproducts promote sustainability by enabling waste recycling, reducing material consumption, and lower carbon emissions [15,16]. Economically, they offer cost-effectiveness by cutting production and transportation costs [15,16]. Mechanically, incorporation of these byproducts can enhance properties like stiffness, tensile strength, density reduction, and durability [17]. For example, incorporating coconut shells in concrete can improve the compressive strength by 34%, while reducing production costs by 15–20%, and incorporating agro-wastes in construction can lead to energy savings of 30% [17]. Sustainable AM has applications in automotive, healthcare, construction, consumer products, electronics, aerospace, and defense sectors [18].

A few studies [6,7,8] have developed composites using wood fibers, such as sawdust, combined with resins for AM applications, including large-scale housing construction. However, uncompressed wood fibers such as those used in these studies have low density, requiring large storage capacity and high transportation costs. As an alternative, wood pellets have been explored for similar composite applications, offering advantages in density, handling, and transportation [10,11,12].

Hemp fibers are natural fibers that have been used extensively in AM applications for reinforcement [19,20,21,22,23,24,25]. Generally, fiber reinforcement has resulted in a 20–30% increase in the mechanical properties, when compared to pure polymers in fused filament fabrication (FFF) applications [9]. Hemp fibers (HF) have also been incorporated as reinforcements to enhance the mechanical properties of wood-based composites and panels [23,26,27]. Specifically, the bending strength of wood-based particleboard was increased by 170%, when reinforced with hemp fibers [27]. Similarly, hemp hurd (HH), a low-value byproduct of HF production, has been used to improve the mechanical properties of similar composites, due to their small particle size and compatibility with polymer matrices [28,29,30,31].

Despite these advancements, a comprehensive evaluation of diverse formulations, including a rigorous cost analysis of the materials, remains lacking for wood–thermoset polymers in AM applications. This study addresses this gap by exploring novel material combinations. Specifically, we investigate the use of wood pellets as an alternative to sifted wood fibers, while incorporating HH as a reinforcement, along with the HF used in prior work [8]. HH has never been demonstrated as a reinforcement for primarily bio-material based additive manufacturing. It was previously observed [8] that low liquid (SS) content significantly increased the motor power required during extrusion; therefore, this study also explores whether substituting a portion of the SS with added water can reduce the power demand while maintaining mechanical strength.

Our objective is to improve the mechanical strength of the composite for construction applications. We hypothesize that the HH used in this study, with its finer particle size, will improve composite strength by enhancing packing density. Smaller particle sizes have previously shown to improve strength in the literature [32,33,34]. This study compares wood pellets against both sifted and unsifted wood fibers to establish the effect of wood particle size and its interaction with HH reinforcement. The study quantitatively compares formulations of three different wood feedstocks based on bulk density, rheological behavior and extrudability, flexural and compression strength, and strength-to-cost ratios with full factorial optimization. By optimizing the formulation based on these factors, this work aims to create and optimize, to our knowledge, the first HH-reinforced wood-based material for sustainable AM, making it more viable for construction and other applications.

## 2. Materials and Methods

### 2.1. Materials

**Figure 1:**
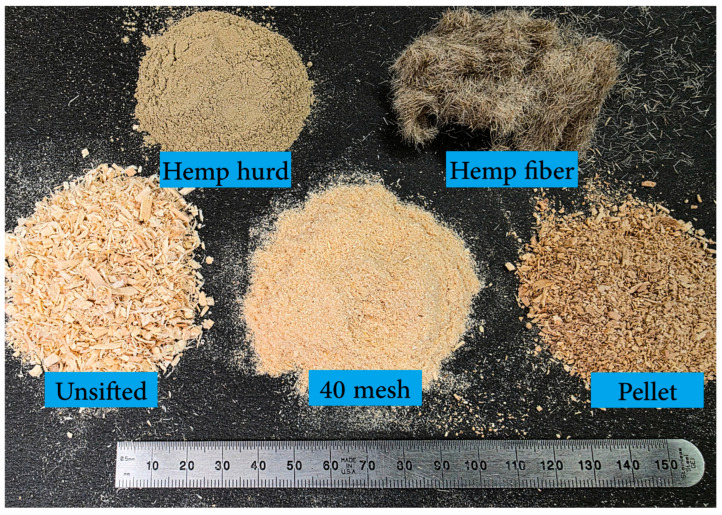
Wood fibers, hemp hurd, and hemp fiber.

The composites consisted of wood fibers (WF), SS, HH, and HF in varying amounts. Three different WF types (WFT) were tested, (1) unsifted WF provided by Plummer Forest Products (Post Falls, ID, USA), referred to as unsifted; (2) the same wood fibers sifted through a 40-mesh sieve, referred to as 40 mesh; and (3) commercial wood pellets (bulk pellets) were purchased through North Idaho Energy Logs [35], which were ground in 100 g batches using a coffee grinder and referred to as pellet. Commercial SS solution (37%) purchased from ThermoFisher Scientific (Pittsburgh, PA, USA) was used as resin. Micronized HH was provided by United Fiber Co. (Savannah, MO, USA) HF , in the form of “Degummed Hemp Sliver” was purchased from Hemptraders.com. The fiber bundles were chopped to 3 mm lengths using an automatic textile ribbon cutter [36]. The solid constituents are shown in Figure 1.

### 2.2. Design of Experiments

The study consisted of preparing composites of various formulations selected based on prior studies and preliminary tests. The factors and levels incorporated in the study are listed in Table 1.

SS fraction (dry weight basis) was determined based on findings from a prior study [8], where 45 wt% SS resulted in a very dry formulation and 50 wt%, 55 wt% were suitable for extrusion. 60 wt% was added as HH is expected to make drier formulations. 50 + 5Water indicating 50 wt% SS and 5 wt% water was also tested to see if water makes the formulation less dry and improves the extrudability and rheology. The HH fraction range (0–15 wt%) was selected as the WF is intended to be the primary fiber, greater fractions were not tested. A fixed 5 wt% HF fraction (3 mm length) was used, which were previously determined as optimal [8].

A full factorial experimental design was implemented, generating a total of 48 distinct formulations. Fractional factorial design was not chosen for this study, as interactions are expected, and such designs are less accurate for categorical factors (such as WFT). Statistical analysis was performed using Minitab (V22), with ANOVA at the 95% confidence interval.

### 2.3. Particle Size Analysis and Bulk Density

Particle sizes for WF were measured using sieve analysis with 10, 16, 30, 40, and 60 mesh screens. HH particles were previously characterized to have an average particle size of 64 µm, with most particles being smaller than 150 µm. The selected mesh sizes were chosen to accommodate all three WFT. For analysis, 200 g of each WF type were passed through the meshes using a standard shaker for 30 min, conducted in triplicate, and the average mass of fibers retained in each mesh was measured to estimate the particle size distribution of the WF.

Bulk densities of WF and HH were measured by calculating the mass of materials, (HH, unsifted WF, 40 mesh WF, bulk pellets, and pellet WF) in a 500 mL container, performed in triplicate. The mass was then divided by the volume to calculate the densities.

### 2.4. Rheology

A DHR-2 Rheometer (TA Instruments, New Castle, DE, USA) was used to characterize the rheological properties of the composite formulations. A 25 mm diameter parallel plate setup was used with serrated plates, for better gripping of the specimens [37]. The composite specimens were prepared with a mass of 3 g (dry basis) to achieve ~5 mm thickness. The thickness was chosen based on the presence of wood flour (WF) particles, up to 2.5 mm in size. Ideally, the gap between parallel plates should exceed 10 times the maximum particle size to ensure accurate rheological measurements [38]. However, due to equipment constraints, a gap of 2 times the largest particle size was selected.

Frequency sweep experiments were performed from 0.01 Hz to 100 Hz at 22 °C with 0.1% strain and 3.0 N axial force control. Experiments were in triplicate and the average complex viscosity (η*) at 1 Hz for each composition was recorded.

### 2.5. Extrusion

For each formulation, a 60 g batch (on a dry basis) was processed. First, WF was weighed and added into a high-capacity blender (Waring 700B, Waring Commercial, CT, USA). HH and HF were then added, and the dry mixture was homogenized for 2 min. SS solution was then incorporated, and the entire composite was then blended for an additional 2 min, with a brief interruption around the 1 min mark to manually scrape down the container walls to ensure uniform dispersion of the components.

The prepared composite mixture was hand-fed into the barrel of a commercial single-screw extruder (RobotDigg, Shanghai, China). The extruder was equipped with a 9.4 mm diameter circular die and a zero-compression auger. The motor speed was fixed at 13 rpm, with the motor having a maximum power rating of 300 W. The barrel was cooled with ice-water via a coiled copper tube.

The power draw of the motor, a direct measure of the shear resistance and force required to push the material, was continuously monitored via the extruder’s digital display. The peak power consumption was recorded and rounded up to the nearest increment of 5 W.

**Figure 2:**
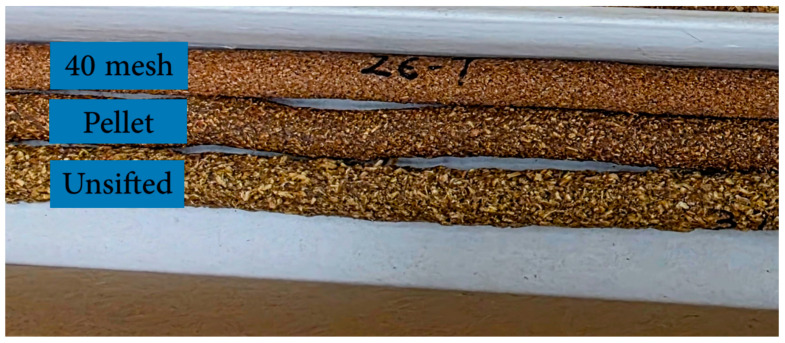
Composite extrudates with 40 mesh, pellet and unsifted WF.

The composite exited the die as a continuous rod and was collected on a cardboard substrate. The nominal extrudate diameter was 9.4 ± 0.2 mm (equal to the die diameter). However, significant variations in the extrudate’s length were observed across the different formulations, suggesting different densities. Immediately after the extrusion, the extrudate along with the cardboard substrate, was transferred to an oven set at 105 °C for a 24 h to cure. Figure 2 shows representative extrudates produced using the different WFT.

After the curing period, the dried rod was removed and allowed to cool to room temperature. The extruded rod was cut to prepare specimens for subsequent density measurement, flexural, and compression testing. All raw materials and cured specimens were stored in sealed, air-tight polyethylene bags throughout the study to maintain a consistent moisture content.

### 2.6. Specimen Density, Flexural, and Compression Strength Testing

The cured composite rods were cut to make five specimens, each 170 mm in length, using a miter saw. The specimens were then weighed and using the nominal diameter (9.4 mm) the density of the cured specimens was calculated.

Three-point flexural testing was carried out on these cylindrical specimens using a Mecmesin MultiTest-dV 2.5 universal testing machine (Sterling, VA, USA). The procedure adhered to ASTM D790 [39] for flexural properties, with necessary modifications for a cylindrical geometry as specified in the ASTM D6109 [40] standard. The support span was set to 150 mm, which is approximately 16 times the specimen diameter. The test was conducted at a crosshead speed of 4 mm/min, which was calculated according to the equations provided in ASTM D790.

Five specimens, each 18.8 mm in length, were cut from the cured extrudate using a miter saw. Compression testing was conducted in accordance with the ASTM D695 [41] standard. The measurements were performed on an Instron 5500R universal testing machine (Norwood, MA, USA) with a 50 kN load cell. The specimens were subjected to axial force at a uniform crosshead speed of 0.1 mm/min. All raw data and resulting calculations were captured and stored using Bluehill Universal material testing software (V4.44).

### 2.7. Cost Analysis

Average strength was calculated as the numerical mean of the flexural and compressive strengths. The material cost of making 1 m of the composite was calculated, which accounts for density variations. The strength-to-cost ratio of each formulation was calculated, to determine the most economical composite formulation. Material costs were determined at the time of writing (2025) from prices published on each supplier’s website based on bulk purchasing quantities (>50 kg). The exception is the cost of raw wood fiber, which was obtained by quotation.

Quantitative calculation excluded all manufacturing costs (e.g., labor and equipment); however, shipping costs are qualitatively discussed.

### 2.8. Microscopy

To understand more about their failure mechanisms, fractured surfaces of composites were examined via SEM following flexural testing. Specimens composed of 55% SS, of each fiber type, and with HH fractions of 5% and 15% were imaged using a Zeiss Supra 35 FEG-SEM (Oberkochen, Germany) with secondary electron detector. Specimens were sputter coated with gold prior to imaging to provide conductivity.

### 2.9. Additive Manufacturing

The optimized formulation, based on the cost, mechanical properties, and extrudability was selected for additive manufacturing demonstration. A simple 3-layer g-code was written manually and exported to Pronterface software (V2.1) on a computer that was connected to a custom-built 3D printer [6].

## 3. Results

### 3.1. Particle Size Analysis and Bulk Density of Constituent Materials

**Figure 3:**
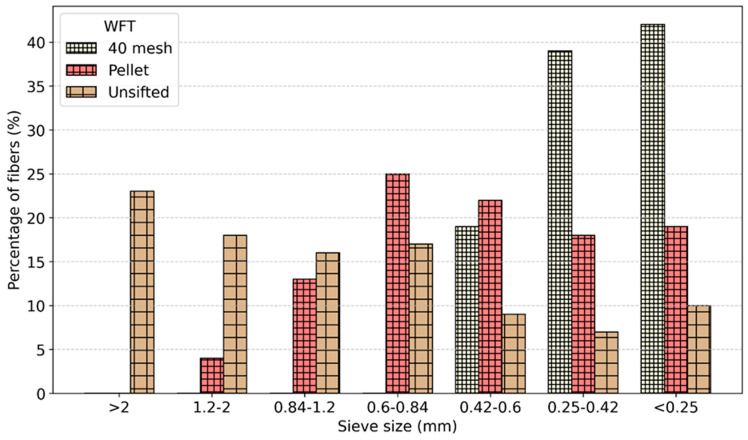
Particle size distribution of WFT determined by sieve analysis.

Sieve analysis for all three WFT are shown in Figure 3. The unsifted WF exhibited the largest particles, with 23% of its fibers exceeding 2 mm. Pellet WF contained no particles larger than 2 mm. When considering all particles greater than 0.6 mm, unsifted WF had the highest proportion, at 57%, followed by pellets WF at 42%. The 40 mesh WF contained no particles larger than 0.6 mm.

**Figure 4:**
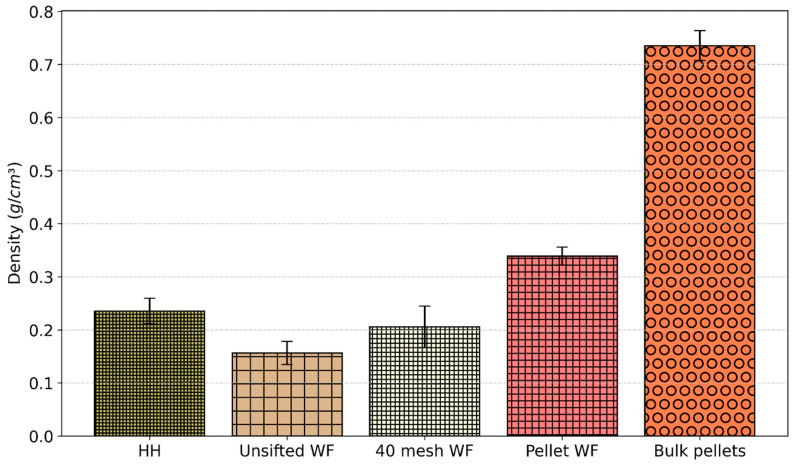
Bulk densities of constituent materials.

Bulk densities of the materials are shown in Figure 4. Bulk pellets had the highest density at 0.74 g/cm^3^. The process of grinding this material into pellet WF resulted in a significant decrease in density, dropping to 0.34 g/cm^3^. Among the WF, pellet WF had the highest density (0.34 g/cm^3^), followed by 40 mesh WF (0.21 g/cm^3^) and unsifted WF (0.16 g/cm^3^).

### 3.2. Rheology and Extrusion

Rheology was conducted on various formulations and the complex viscosity (η*) data at 1 Hz was analyzed. ANOVA indicated that η* was significantly affected by SS and HH fractions (*p* < 0.01), but there was no direct effect of WFT (*p* > 0.07). There was a significant interaction between HH and SS fractions (*p* < 0.01), and between HH fractions and WFT (*p* < 0.01). The contribution of each factor towards viscosity was also measured. SS fraction contributed the largest (38.9%), followed by the interaction of HH and SS fractions (24.0%), and HH fraction (11.2%). Other factors and interactions contributed less than 10% each.

**Figure 5:**
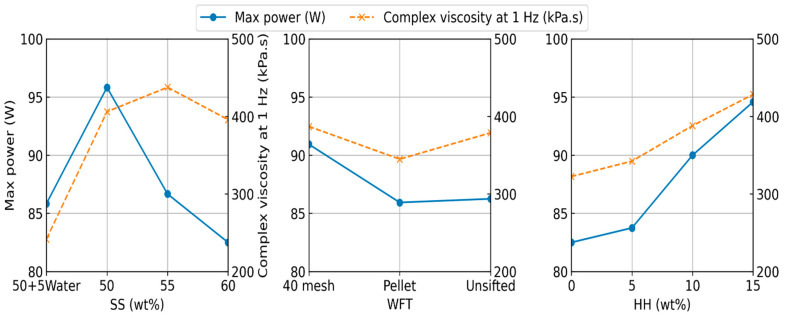
Main effects for complex viscosity and motor power for composite extrusion.

Power without any extrudate (baseline motor power) was approximately 65 W. Required power increased gradually as the mixture was being fed for extrusion. All the formulations extruded, however, certain formulations took longer and stalled for brief periods of time and then started flowing again. ANOVA results demonstrated that HH fraction, SS fraction and WFT significantly changed the maximum motor power (*p* < 0.01). The interaction of HH and SS fractions was also significant (*p* < 0.01); no other factors were significant (*p* > 0.06). SS fraction contributed the most to maximum motor power at 32.9%, HH fraction at 29.5%, and the interaction of HH and SS fractions contributed 12.0%. Other factors contributed less than 10% each. The main effects for viscosity and motor power are plotted in Figure 5. Interaction plots are shown in Supplementary Materials Figures S1–S6.

### 3.3. Specimen Density, Flexural and Compression Strength Testing

For density, WFT, HH, and SS fractions, along with the interaction between HH and SS fractions were all significant factors (*p* < 0.01). All other interactions were insignificant (*p* > 0.2). WFT contributed to 57.9% of the difference in density, followed by HH fraction at 21.1%. All other factors contributed less than 10% each.

For flexural strength, ANOVA indicated that WFT, HH, and SS fractions, along with the interaction between HH fractions and WFT, were all significant factors (*p* < 0.01). All other interactions were insignificant (*p* > 0.1). WFT contributed to most of the difference in flexural strength, at 62.6%, other factors contributed less than 10% each.

**Figure 6:**
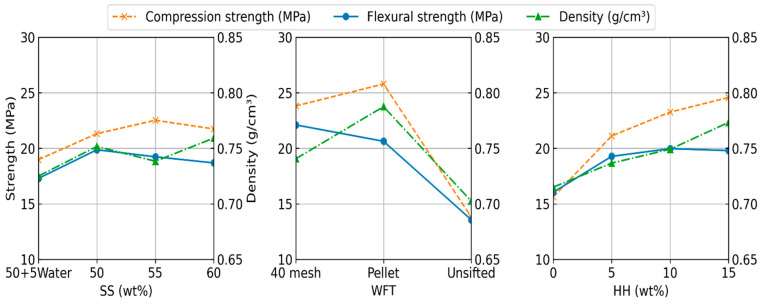
Main effects for density, flexural, and compression strengths for cured composite rods.

**Figure 7:**
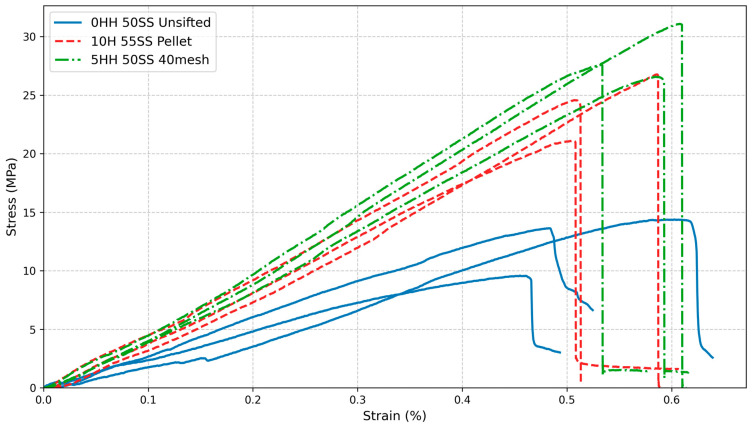
Typical stress–strain curves for flexural tests.

HH and SS fractions along with WFT, and interactions between SS fraction and WFT significantly (*p* < 0.01) affected the compression strength. WFT accounted for 55.3% of the variation, HH fraction accounted for 21.2%, all other factors and interactions contributed less than 10% each. The main effects for density, flexural and compression strengths are plotted in Figure 6. Interaction plots are shown in Supplementary Materials Figures S7–S15. Typical Stress–strain curves for the flexural tests are shown in Figure 7.

A complete summary for ANOVA of viscosity, motor power, specimen density, and flexural and compression strengths are presented in Table 2.

### 3.4. Cost Analysis

The costs of the various raw materials used to make the composites were obtained. All amounts are in USD and were obtained in July 2025. Among WFT, the lowest cost was that of unsifted WF at around 0.11 $/kg. Commercial pellets were priced at 0.32 $/kg. The cost of 40 mesh WF was 0.85 $/kg. HH and HF both had a similar cost of about 6.61 $/kg. The cost of SS was 3.79 $/kg. The material costs are summarized in Table 3.

Based on these values, the average material cost for preparing a 60 g (dry basis) composite batch was calculated to be $0.39 (SD $0.03). Strength-to-cost ratios ranged from 21.3 MPa/$ for the formulation with unsifted WF, 0 wt% HH, and 60 wt% SS to 86.6 MPa/$ for the formulation with 40 mesh WF, 5 wt% HH, and 50 wt% SS.

### 3.5. Scanning Electron Microscopy

**Figure 8:**
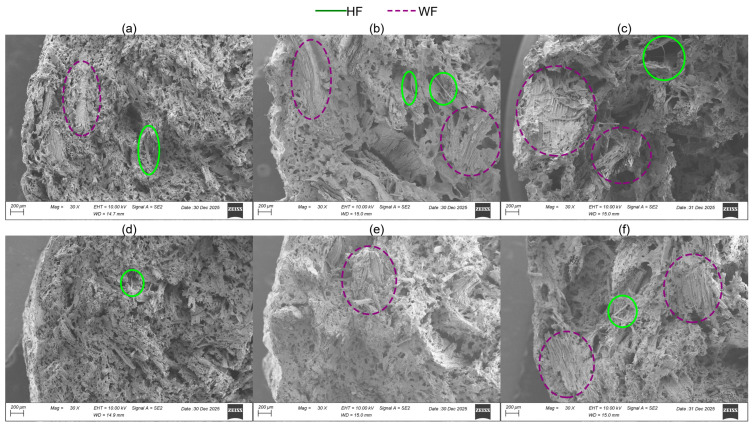
SEM micrographs for formulations containing 55 wt% SS, and (**a**) 5 wt% HH, 40 mesh WF; (**b**) 5 wt% HH, pellet WF; (**c**) 5 wt% HH, unsifted WF; (**d**) 15 wt% HH, 40 mesh WF; (**e**) 15 wt% HH, pellet WF; (**f**) 15 wt% HH, unsifted WF.

Composites containing unsifted WF exhibited the largest voids, followed by those with pellet WF. In contrast, the composites containing 40 mesh WF displayed a higher number of voids, but of smaller dimensions. It was also observed that increasing the HH content resulted in fewer voids, although this effect was less significant than the influence of the WFT. Several components have been labeled in the micrographs of Figure 8.

## 4. Discussion

**Figure 9:**
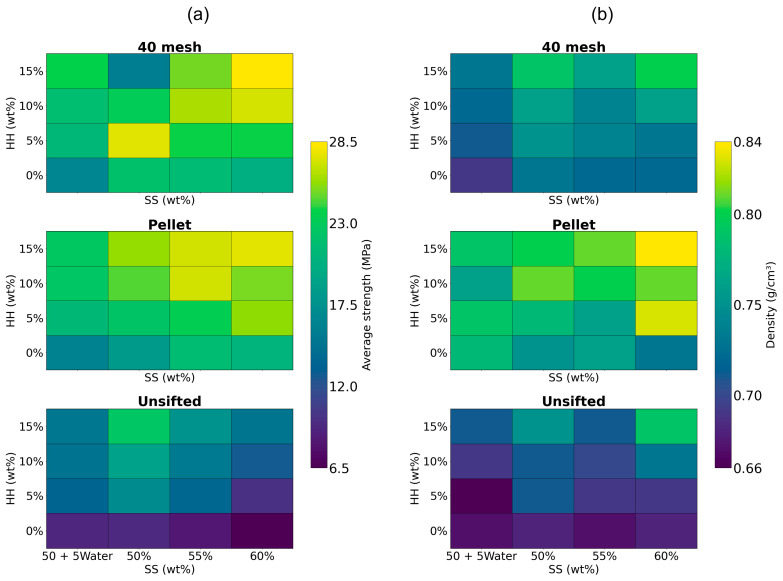
Contour plots of composites for (**a**) average strength and (**b**) average density.

Figure 9a,b show the contour plots for the average strength and density, respectively. The mechanical strength of the cured composite was primarily influenced by the particle size (HH and WF), with finer particle sizes generally yielding higher strength due to improved packing. This finding aligns with our hypothesis and agrees with similar findings in the literature [32,33,34]. This trend is evident as both flexural and compressive strength increased with HH fraction. An exception to this increase was observed in the 40 mesh WF plot. At high HH fractions (10, 15 wt%), at 50 wt% SS, and at 15 wt% HH, at 55 wt% SS, the average strength decreased. This reduction could be attributed to processing difficulties. As the HH content increased, material was qualitatively noted to be harder to mix, resulting in non-homogenous mixing. The motor power required for extrusion rose significantly and extrusion was irregular and slower. The motor frequently stalled and the material had to be forced into the extruder barrel. This could also be linked to a lack of resin, causing reduced binding between particles. Regardless, these challenges demonstrate that processing limitations can lead to sub-optimal or reduced mechanical strength.

Flexural strength was generally highest for the 40 mesh WF (22.1 MPa), followed by the pellet WF (21.0 MPa). However, compression strength was generally higher for pellet WF (25.4 MPa) than the 40 mesh WF (24.6 MPa). This could be attributed to the processing of pellets during which lignin is plasticized and helps bind the wood pellets [42]. Lignin is known to improve compressive strength in many composites [20,43,44,45,46]. In addition, the higher density of pellet WF could also contribute to the compression strength of the composite by increasing its overall density. Unsifted WF consistently showed significantly lower strengths compared to the other two (13.8 and 13.7 MPa for flexural and compression strengths, respectively). This is directly attributed to the presence of substantially larger particles (>2 mm), which can disrupt the composite matrix and lead to the formation of voids, weakening the material. Average strength was similar across SS fractions. However, when water was substituted for SS, the average strength was reduced from 20.9 MPa at 55 wt% to 18.2 MPa at 50 + 5Water. The highest average strengths were at 60 wt% SS, 15 wt% HH, for 40 mesh (28.5 MPa) and pellet WF (27.5 MPa). These values represent an increase of 81.5% and 75%, respectively, over the average strength of the original 40 mesh WF—50 wt% SS composite (15.7 MPa) prepared and tested under similar conditions [8].

The density of the composites with the various WFT followed the densities of the constituent WF, with composites with pellet WF having the highest density (0.79 g/cm^3^), followed by 40 mesh WF (0.74 g/cm^3^) and unsifted WF (0.7 g/cm^3^). Higher fractions of HH generally led to higher densities, ranging from and 0.72 g/cm^3^ at 0 wt% to 0.77 g/cm^3^ at 15 wt%. Adding water to the formulation as a substitute for SS, reduced density from 0.74 g/cm^3^ at 55 wt% to 0.72 g/cm^3^ at 50 + 5Water. This is expected because water completely evaporates during the curing process, whereas SS leaves a 37% residue. The densities observed were lower than the average density (0.89 g/cm^3^) of the original 40 mesh WF—50 wt% SS formulation [6]. This can be attributed to the different curing methodologies: while the original formulation was cured at ambient conditions, curing at 105 °C likely evaporated the composite’s entire water content.

**Figure 10:**
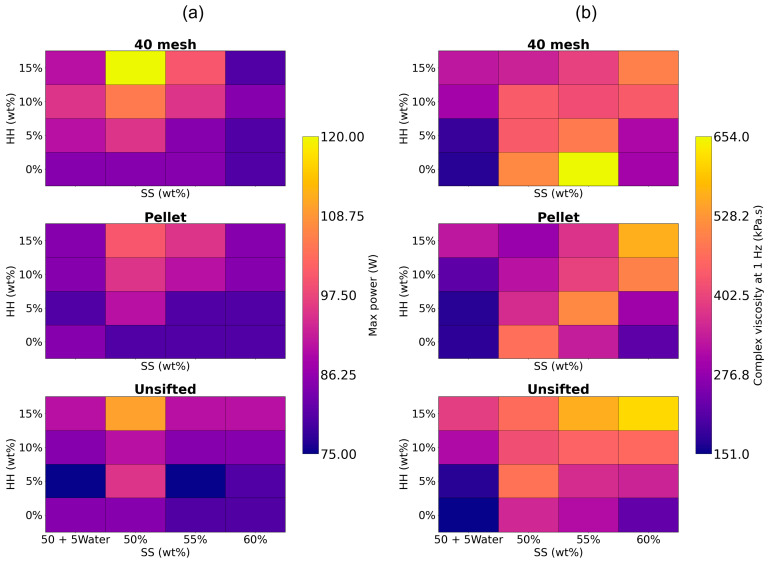
Contour plots for wet composite blends of (**a**) maximum motor power and (**b**) average complex viscosity.

Contour plots for maximum motor power during extrusions and the average complex viscosity for the formulations are shown in Figure 10a,b, respectively. Motor power was highest (95.8 W) at 50 wt% SS and decreased as the SS fraction was increased (86.7 and 82.5 W at 55 wt% and 60 wt% respectively). This is expected, as increasing the SS fraction increases the liquid content of the composite, reducing inter-particle friction and making the overall extrusion process easier. The substitution of water for SS also yielded a comparable reduction in motor power (85.8 W) due to the same mechanism. Motor power increased with increasing HH fractions (from 82.5 W at 0 wt% to 94.6 W at 15 wt%) due to the relatively small particle size of HH, which significantly increases the total fiber surface area. This increased surface area requires more SS solution to wet the particles, effectively making the composite formulation drier [33]. Extrusion power was high for 40 mesh WF (90.9 W) but was lower for pellet WF (85.9 W) and unsifted WF (86.2 W), which is similarly explained by the finer particle size for 40 mesh WF.

It was qualitatively observed that formulations with a maximum motor power exceeding 90 W often had longer extrusion times, and the composite was more difficult to mix, often resulting in non-homogenous mixtures. Feeding the mixture into the extruder barrel was more difficult. Furthermore, the motor overheated after 30 min of extrusion.

Viscosity was high at low SS/low HH (443.3 kPa.s); and high SS/high HH fractions (557.7 kPa.s), and along a diagonal path between these two points. Complex viscosity was low at low SS/high HH (364 kPa.s) and high SS/low HH fractions (246.3 kPa.s). This behavior is attributed to two opposing mechanisms related to solid content and particle size: when the solid content is too low and/or coarse, the SS solution acts primarily as a lubricant, wetting the solid particles and promoting flow and thus reducing viscosity. When the solid content is very high and/or fine, the measured viscosity of the dry composite may be artificially lowered due to wall slip or surface breakdown of the fibers near the plate [47,48]. While the use of serrated plates is expected to reduce wall slip [37], the rheological properties of different fractions should be validated using other methodologies.

The measured viscosity values at high solid fractions, while potentially subject to measurement artifacts such as wall slip [47,48], are still valuable for establishing an optimum material fractions range. For very high fractions of solids and finer particle sizes, the motor power increased significantly (>90 W), whereas for very low solid content and coarser particle sizes, flexural and compression strengths decreased. An optimal balance between extrudability and mechanical strength was typically achieved at viscosities exceeding 400 kPa·s for this specific test equipment. These findings align with a prior study [8], in which it was reported that the formulations with 45 wt% SS had low viscosities and high motor power.

For wood–SS-based composites, parallel plate rheological testing is proposed as an effective and efficient initial screening criterion for determining feasibility of the formulation, for AM applications. This methodology offers substantial benefits, as rheological analysis requires only 3 g (dry basis) of material, compared to the 60 g required for full extrusion and mechanical testing, resulting in significant savings in both resources and experimental time.

**Figure 11:**
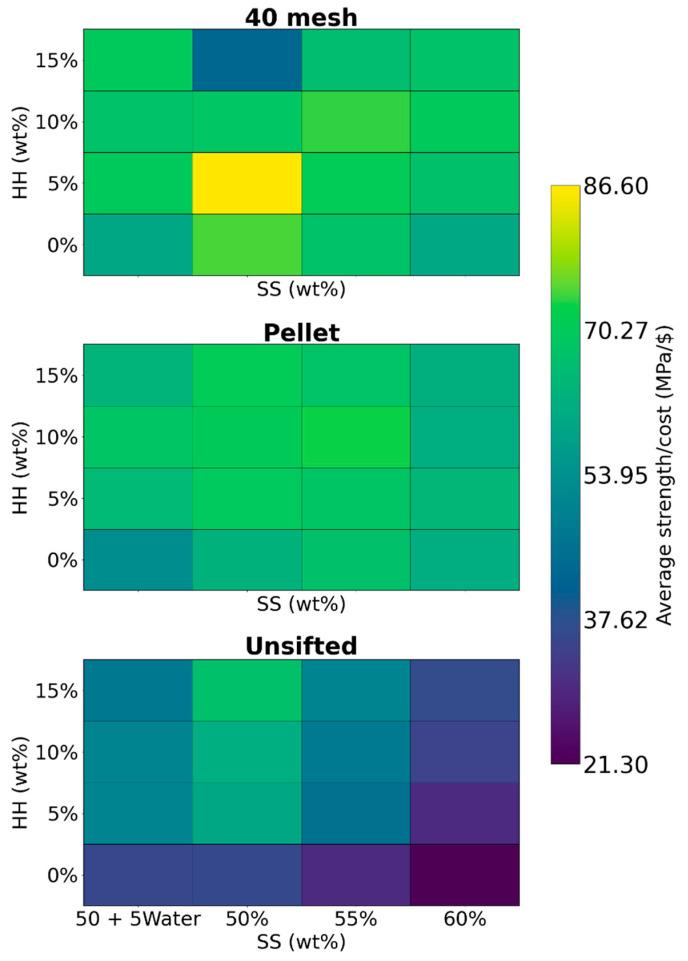
Contour plots for average strength-to-cost ratio.

Contour plots for the strength-to-cost ratio are presented in Figure 11. The formulation with 40 mesh WF, 5 wt% HH, and 50 wt% SS showed the highest ratio at 86.6 MPa/$. However, this formulation required a maximum motor power of 95 W during extrusion. Among the formulations that had lower motor power (≤90 W), the highest strength-to-cost ratio (74.5 MPa/$) was with 40 mesh WF, 50 wt% SS, and no HH. The formulation with pellet WF, 55 wt% SS, and 10 wt% HH had a slightly lower strength-to-cost ratio of 73.0 MPa/$. The average strengths of these formulations were 21.9 MPa and 27.1 MPa, respectively. However, due to the lower SS content used, absence of HH, lower cost of 40 mesh WF compared to pellet WF, and the lower density of the composite, the overall costs of the former formulation are substantially lower, improving its strength-to-cost ratio. To compare with polylactic acid (PLA), a material commonly used in additive manufacturing, the average mechanical strength (flexural and compressive) of PLA is approximately 57.2 MPa [49]. The cost of PLA filament, based on the most widely purchased filament on Amazon.com, is approximately $28/kg [50], while PLA pellets are priced at approximately $7.8/kg [51]. For specimens of comparable dimensions to those in this study, this yields an average strength-to-cost ratio of 23.7 MPa/$ for PLA filament and 84.9 MPa/$ for PLA pellets, respectively. This shows that the formulations in this study are comparable to commercial PLA pellets, although the applications are different.

Using wood pellets offers several logistical and environmental advantages over wood fibers. Wood pellets are much more readily available, as evidenced by the local supermarket price of 0.32 kg/$ in Moscow, Idaho, USA. Wood fibers, in the form of sawdust, are not as easily available and often have to be shipped from sawmills directly. The low density of unpelleted wood fibers (0.16–0.24 g/cm^3^) necessitates significantly higher transportation costs, requiring larger trucks/trailers. The total cost of transporting wood fibers is estimated to be three to four times higher than transporting pellets [12]. Utilizing pellets offers a minimum potential reduction of 25% in the combined material and shipping costs [10]. Furthermore, a life cycle assessment showed that, when compared to wood fibers, pellets had lower global warming potential [11]. Pellets offer easier storage and handling, and they are also easily broken down to an adequate particle size using a simple coffee grinder. Due to these factors, the formulation containing pellet WF, 55 wt% SS and 10 wt% HH with a slightly lower strength-to-cost ratio of 73.0 MPa/$ is preferred. If extrudability requirements change (e.g., due to a different motor), the formulation can be readily adjusted to contain 5 wt% HH or 15 wt% HH, which decreases or increases motor power, respectively. Notably, these alternatives still achieve fairly high strength-to-cost ratios of 68.7 MPa/$ and 68.4 MPa/$, respectively, maintaining excellent cost-effectiveness.

**Figure 12:**
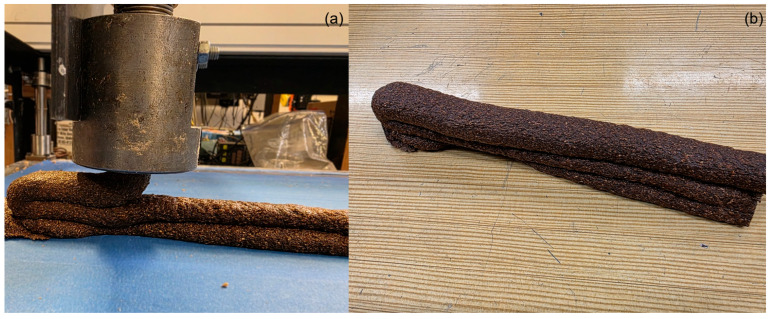
Demonstration of the optimized formulation’s printability: (**a**) in-process view, and (**b**) the as-printed part.

The optimized formulation containing pellet WF, 55 wt% SS, 10 wt% HH was used to demonstrate additive manufacturing with a simple three-layer print, shown in Figure 12.

These results establish a practical framework for cost-effective AM materials, reducing wood waste. However, the study has limitations, including qualitative assessments of processing challenges and potential artifacts in rheological property measurements, which require improvement. Findings should be validated on a larger scale using AM equipment. Processing issues, such as inconsistent mixing, motor stalling, and overheating could be mitigated by using a higher-torque motor and twin-screw extrusion. The impact of moisture, ambient temperature, and humidity on material performance should be tested. Microstructure analysis via SEM would provide better understanding and validation of the material behavior.

Future work should prioritize testing the materials for manufacturability and long-term durability in outdoor conditions. Interlayer bonding strength and the mechanical properties of multi-layer parts should also be studied. A detailed cost analysis that includes equipment, processing, labor, and energy expenses should be conducted along with life-cycle assessments. Recyclability of the materials should be investigated to improve sustainability. Alternative resins should be explored for improved biodegradability and reduced material costs.

## 5. Conclusions

This study optimized wood–sodium silicate composites reinforced with hemp hurd (HH) and hemp fibers (HF) for additive manufacturing (AM). Rheological analysis showed that sodium silicate (SS) and HH fractions significantly influenced complex viscosity, with key interactions leading to decreased viscosity in overly dry or wet formulations. Higher viscosities (>400 kPa·s) indicated a strong balance between strength and extrudability, serving as a resource-efficient initial screening tool for wood–SS composites. Extrusion tests revealed that motor power increased in drier formulations. High motor power (>90 W) often led to processing and extrusion challenges. Mechanical strength tests confirmed that finer particle sizes increased flexural and compression strengths. Cost analysis revealed optimal formulations based on strength-to-cost ratios, logistics, and processability, with pellet wood fibers at 55 wt% SS and 10 wt% HH yielding the top value of 73.0 MPa/$.

HH was used for the first time to reinforce wood-based composites for AM. A rigorous full-factorial optimization and strength to cost analysis highlights this important aspect of large-scale printing. Ultimately, the formulations developed in this study significantly enhance the strength, feasibility, and sustainability of wood–SS composites, while ensuring cost-effectiveness for AM applications. These optimized composites hold potential for scalable AM in affordable housing and infrastructure.

## Figures and Tables

**Table 1:** Factors and levels for composite formulation.

Factor	Levels/Values
WFT	Unsifted, 40 mesh, pellet
SS fraction (wt%)	50, 55, 60, 50 + 5Water
HH fraction (wt%)	0, 5, 10, 15
HF fraction (wt%)	Fixed at 5 (3 mm length)
WF fraction (wt%)	Balance to 100%

**Table 2:** *p*-values and percentage contributions towards the responses for composite rod formulations and properties.

Variables	Metric	Responses				
		Power	Viscosity	Density	Flexural Strength	Compression Strength
SS	*p*-Value	0	0	0	0.026	0.008
	% Contribution	32.9	38.9	8.2	4.0	3.5
HH	*p*-Value	0	0	0	0	0
	% Contribution	29.5	11.2	21.1	8.1	21.2
WFT	*p*-Value	0.003	0.073	0	0	0
	% Contribution	6.1	2.3	57.9	62.6	55.3
SS × HH	*p*-Value	0	0	0.003	0.506	0.304
	% Contribution	12.0	24.0	4.1	0.9	0.9
SS × WFT	*p*-Value	0.054	0.45	0.234	0.101	0
	% Contribution	4.9	2.4	1.9	4.5	8.9
HH × WFT	*p*-Value	0.657	0	0.603	0.002	0.171
	% Contribution	0.4	9.7	0.2	5.7	0.9

**Table 3:** Costs of materials.

Material	Cost in USD ($/kg)
Unsifted WF	0.11
Pellet WF	0.32
40 mesh WF	0.85
HH	6.61
HF	6.61
SS	3.79

## Data Availability

The original contributions presented in this study are included in the article/Supplementary Materials. Further inquiries can be directed to the corresponding author.

## Supplementary Materials

The following supporting information can be downloaded at: https://www.mdpi.com/article/10.3390/ma19020357/s1, Figure S1: HH × SS interaction for complex viscosity; Figure S2: HH × WFT interaction for complex viscosity; Figure S3: SS × WFT interaction for complex viscosity; Figure S4: HH × SS interaction for max motor power; Figure S5: HH × WFT interaction for max motor power; Figure S6: SS × WFT interaction for max motor power; Figure S7: HH × SS interaction for compression strength; Figure S8: HH × WFT interaction for compression strength; Figure S9: SS × WFT interaction for compression strength; Figure S10: HH × SS interaction for flexural strength; Figure S11: HH × WFT interaction for flexural strength; Figure S12: SS × WFT interaction for flexural strength; Figure S13: HH × SS interaction for density; Figure S14: HH × WFT interaction for density; Figure S15: SS × WFT interaction for density.
