# Supplementary material for: Optimizing Wood–Hemp–Sodium Silicate Composites for Strength, Extrudability, and Cost in Additive Manufacturing Applications
Source: Materials (Basel). 2026 Jan 16;19(2):357. doi: 10.3390/ma19020357 (PMC12843062; doi:10.3390/ma19020357)
Supplement: Supplementary file 1 [file materials-19-00357-s001.zip › materials-4004047-supplementary.pdf]

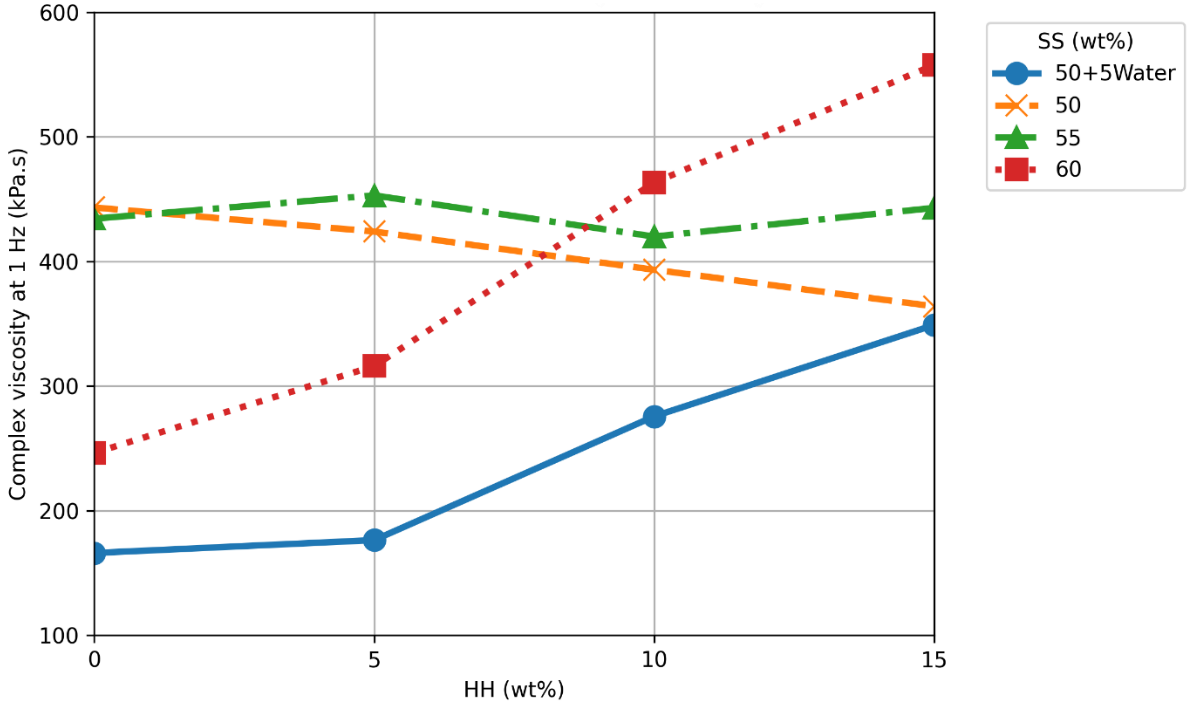

Figure S1: HH × SS interaction for complex viscosity.

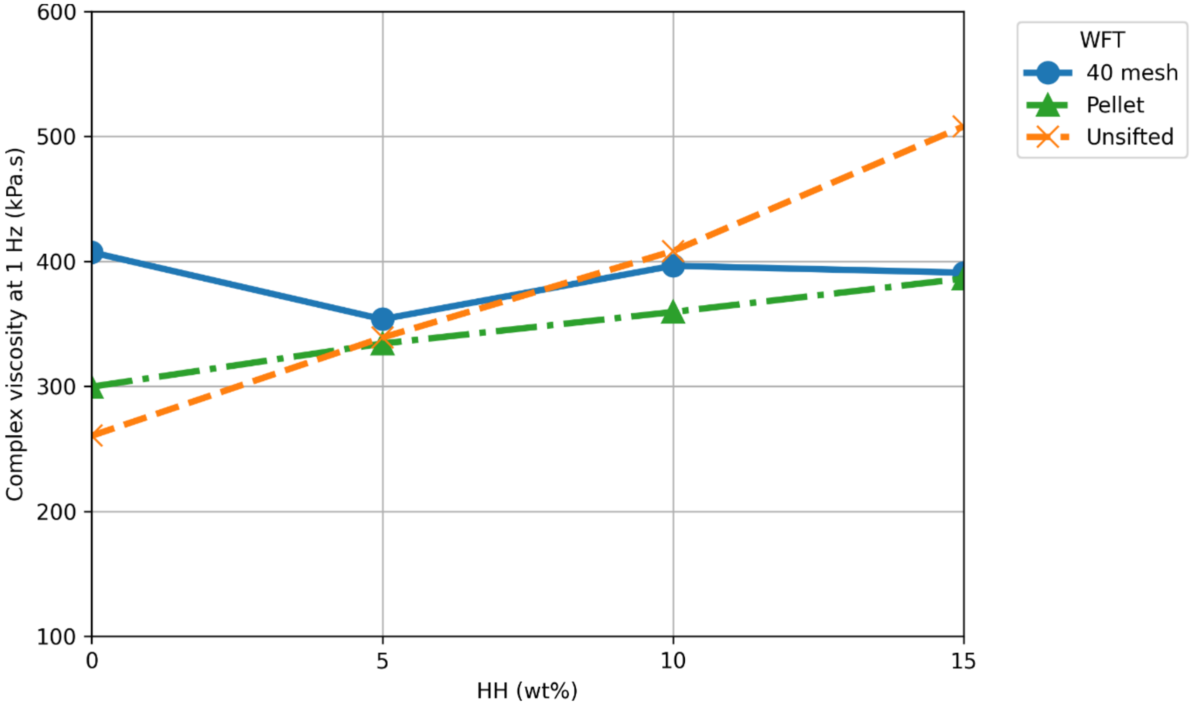

Figure S2: HH × WFT interaction for complex viscosity.

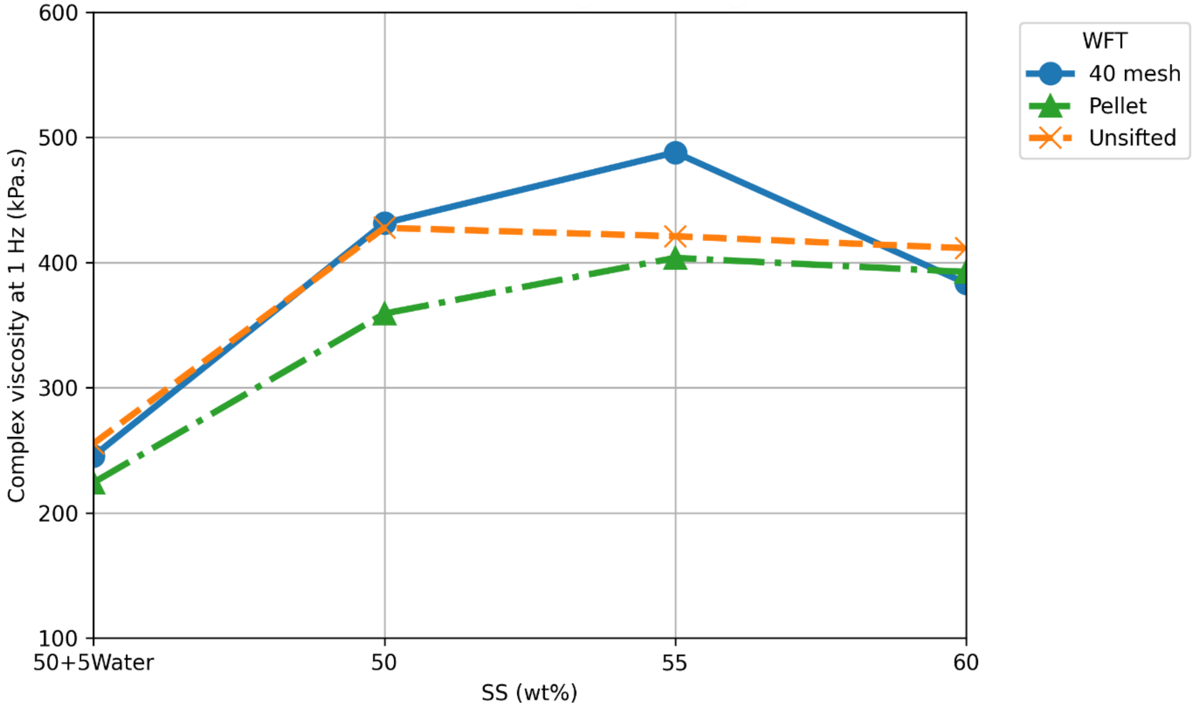

Figure S3: SS × WFT interaction for complex viscosity.

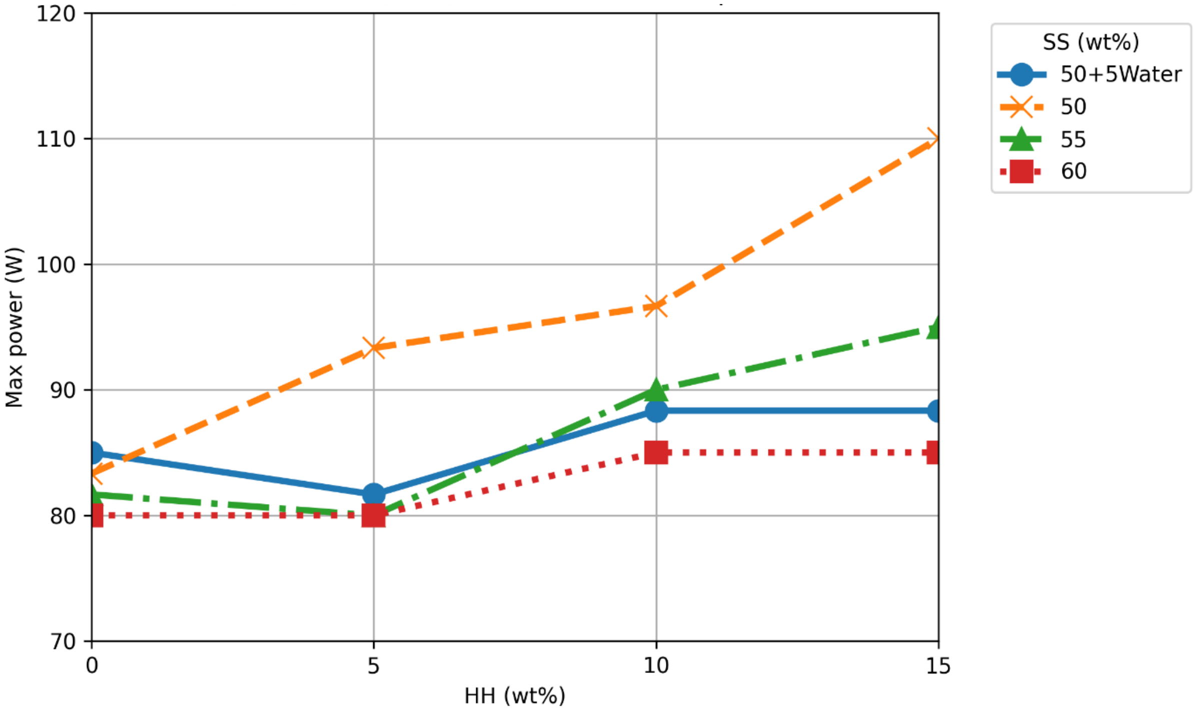

Figure S4: HH × SS interaction for max motor power.

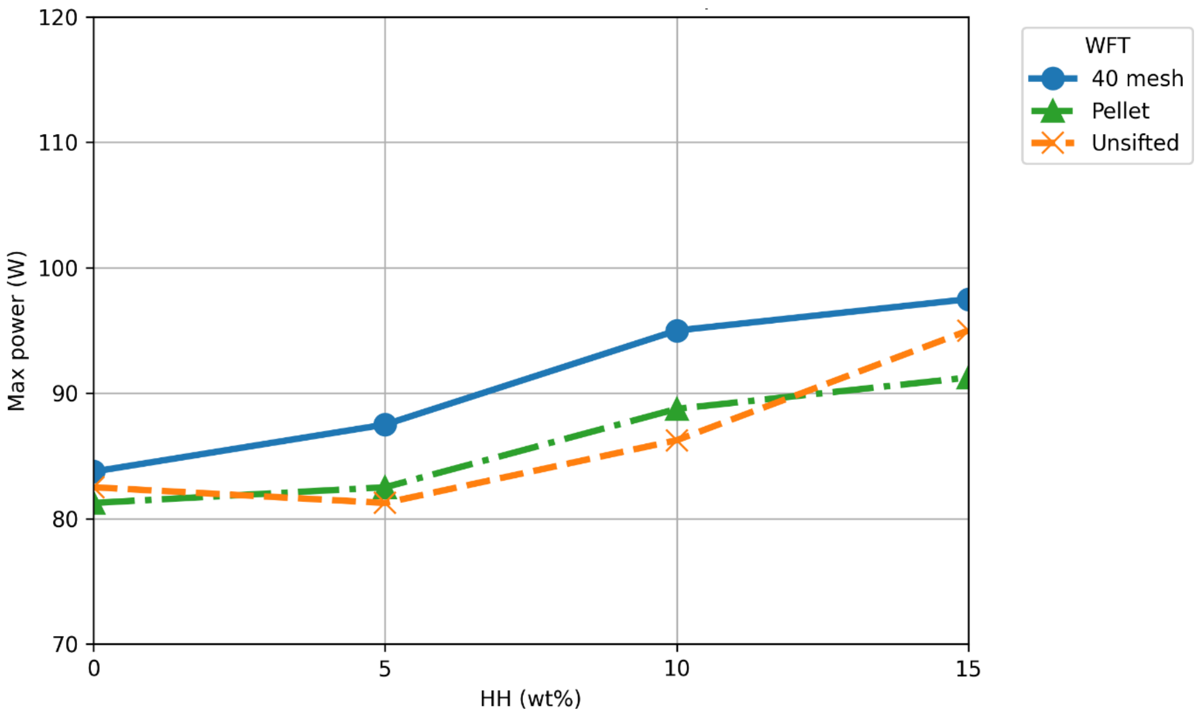

Figure S5: HH × WFT interaction for max motor power.

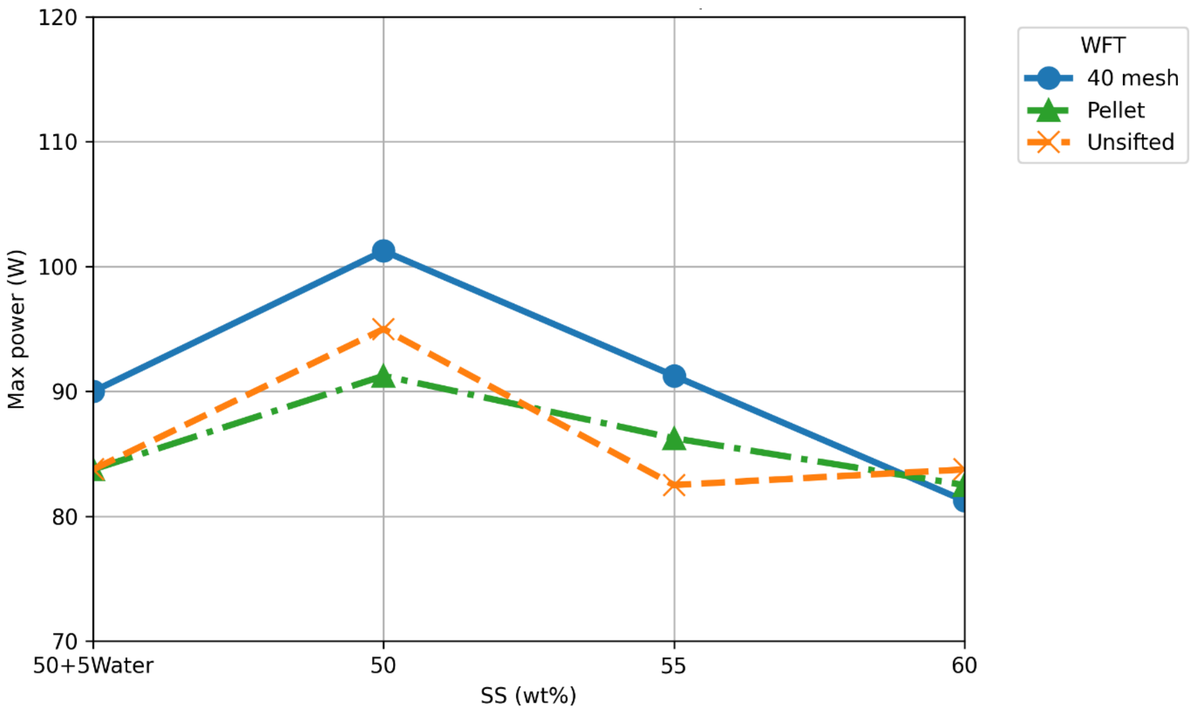

Figure S6: SS × WFT interaction for max motor power.

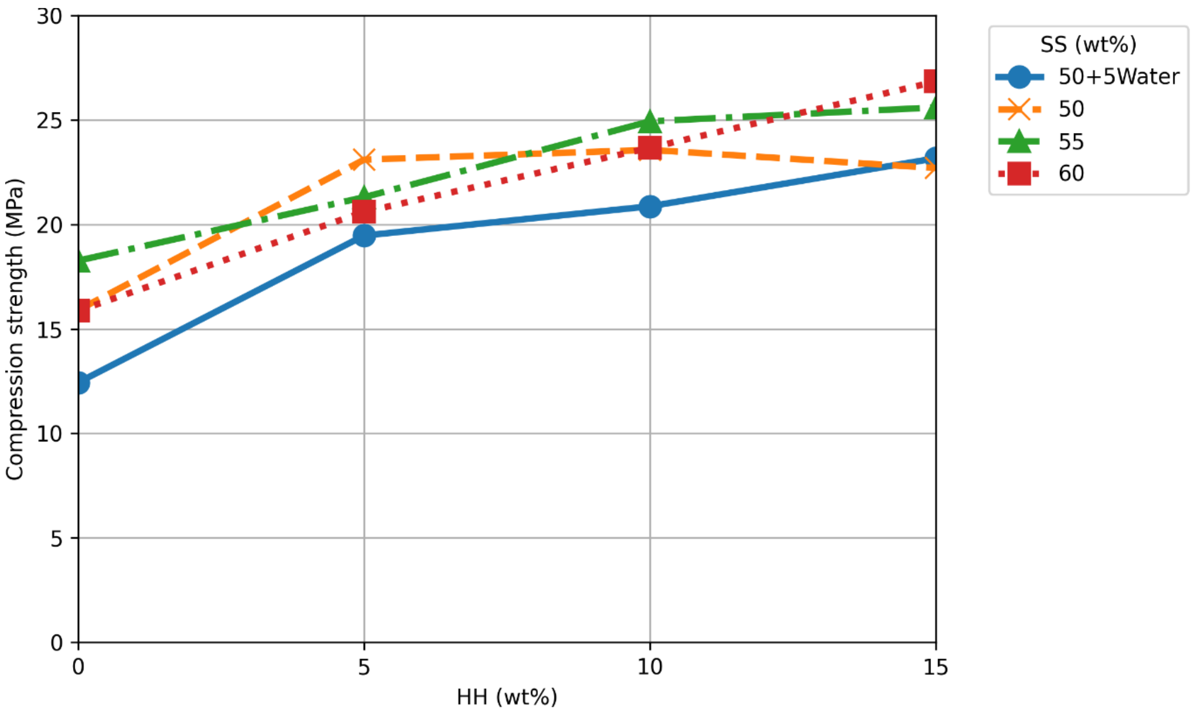

Figure S7: HH × SS interaction for compression strength.

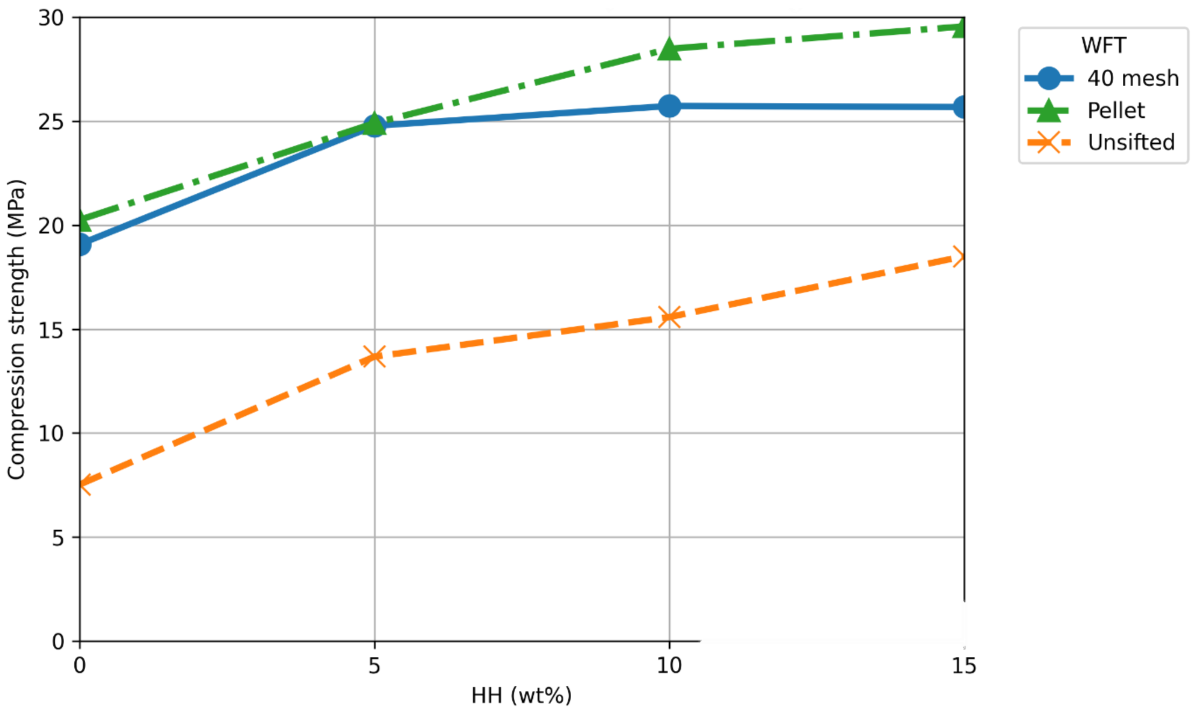

Figure S8: HH × WFT interaction for compression strength.

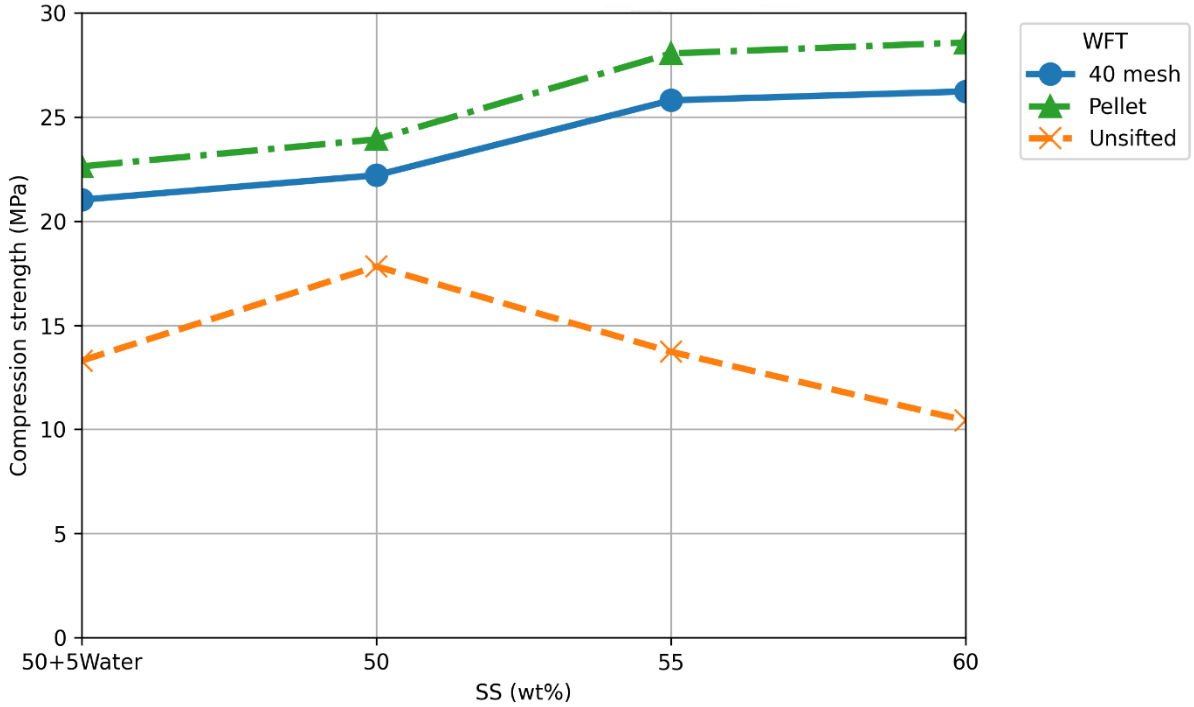

Figure S9: SS × WFT interaction for compression strength.

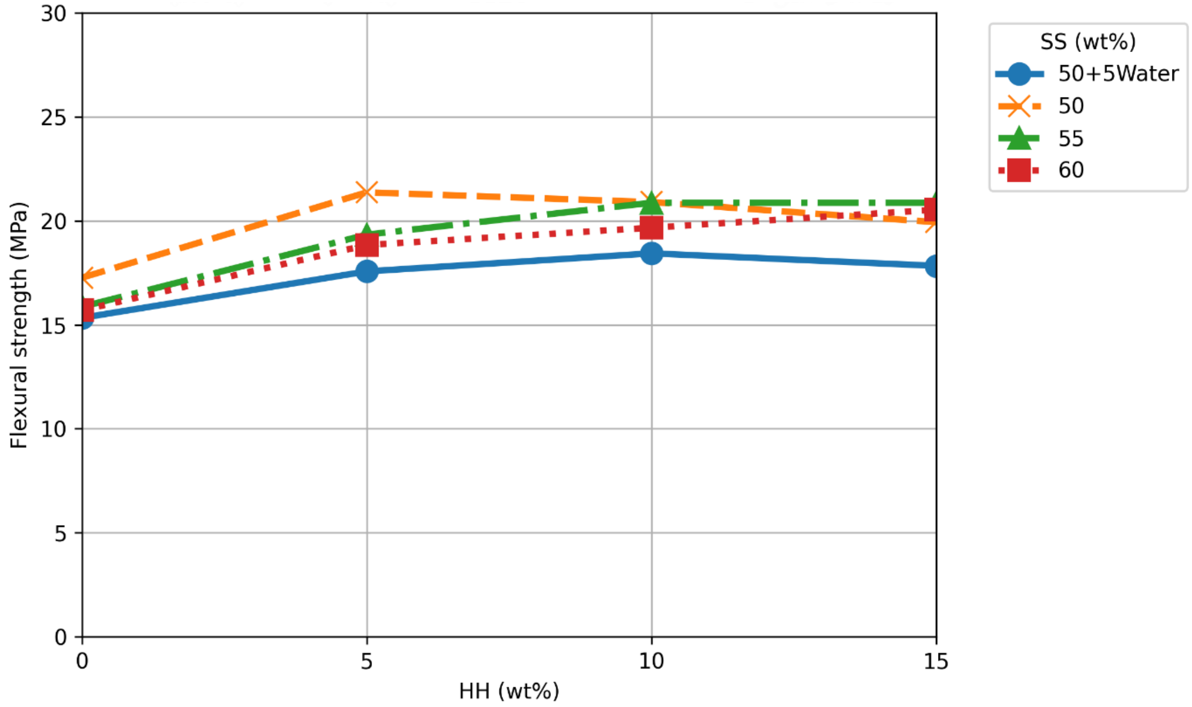

Figure S10: HH × SS interaction for flexural strength.

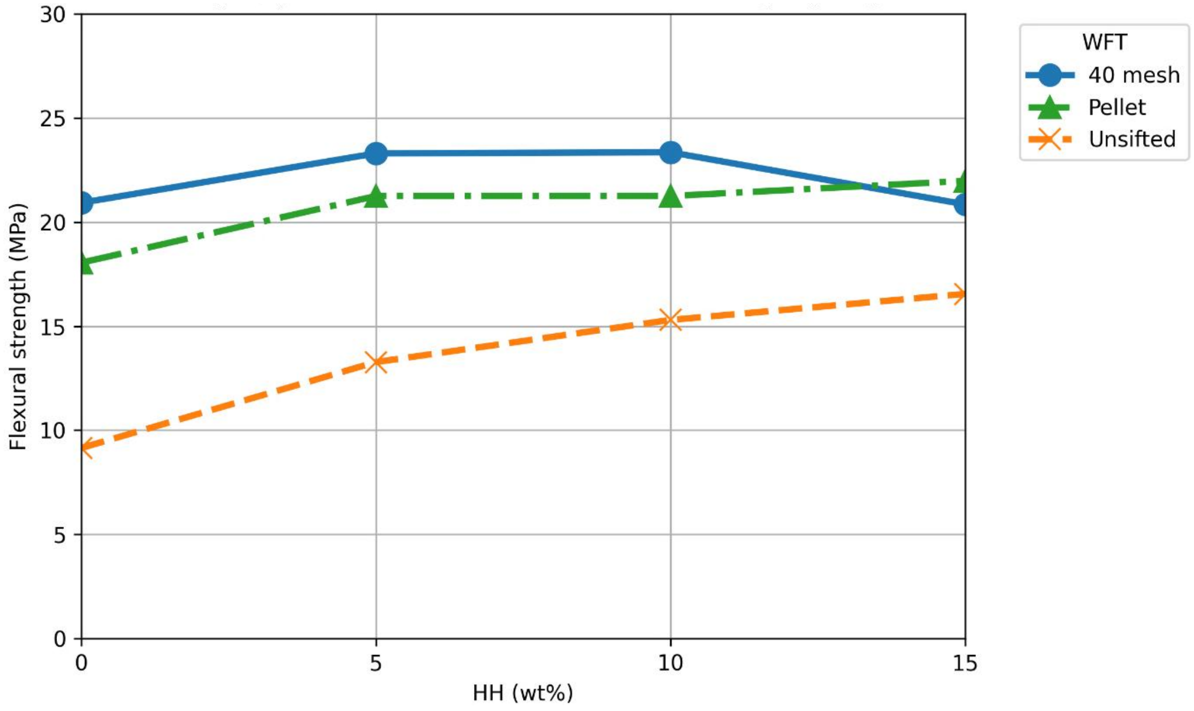

Figure S11: HH × WFT interaction for flexural strength.

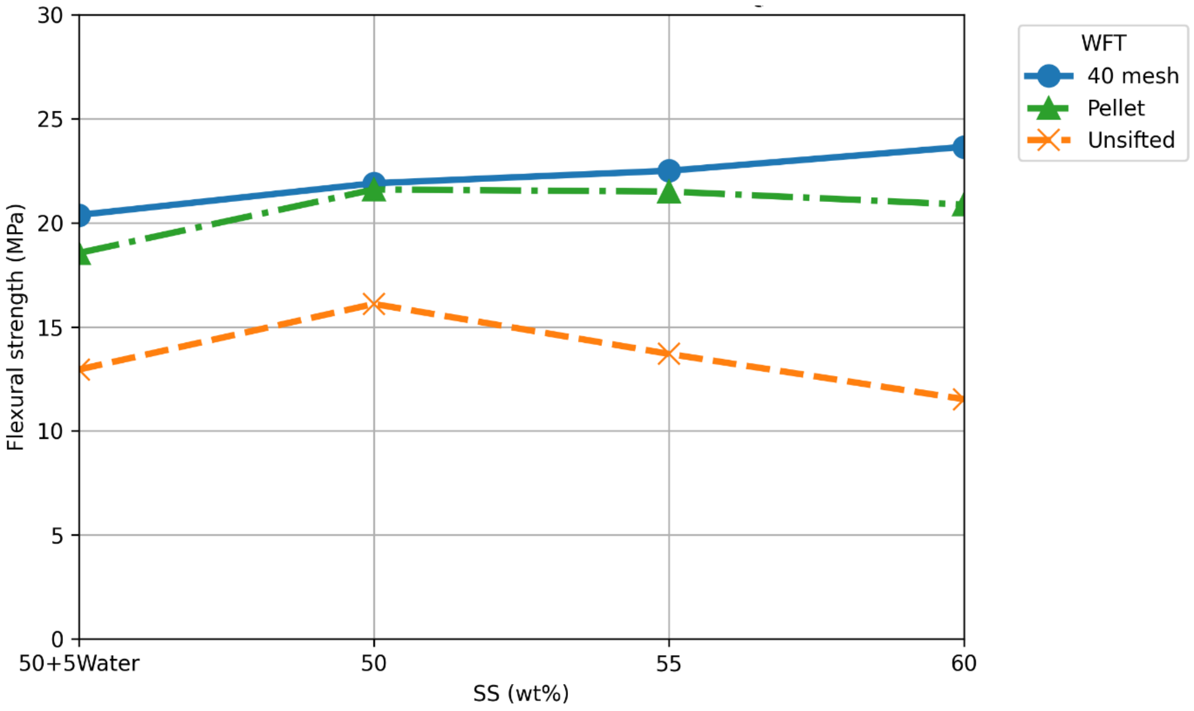

Figure S12: SS × WFT interaction for flexural strength.

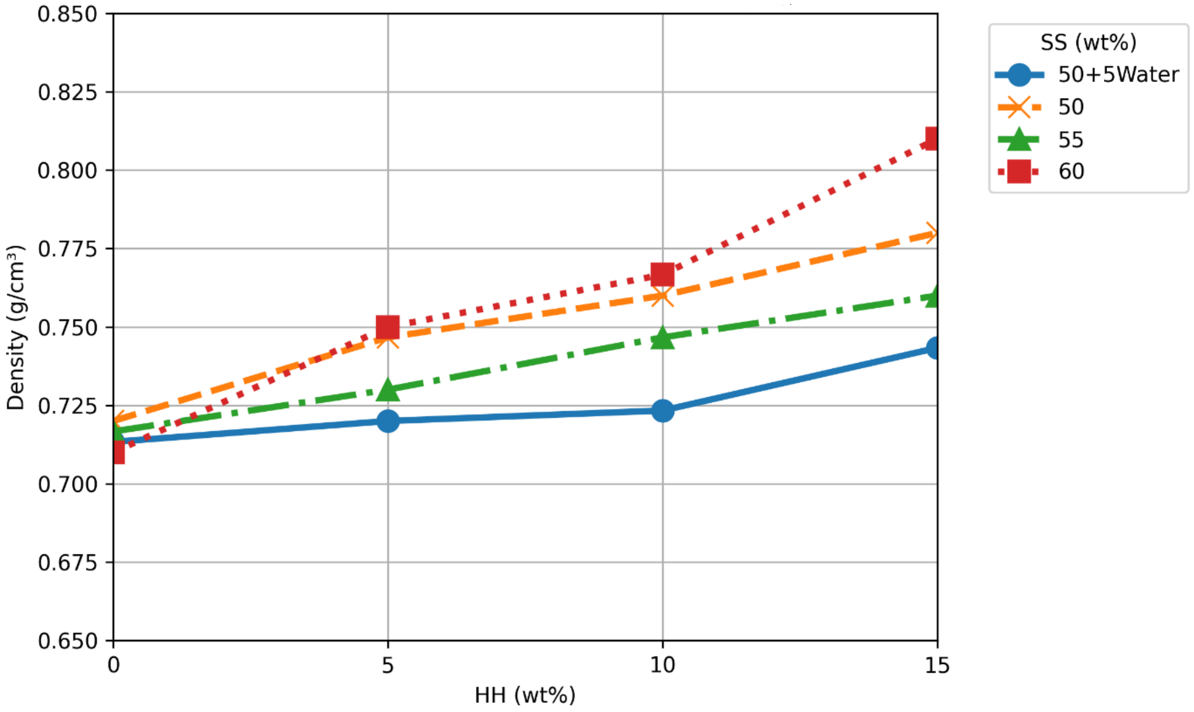

Figure S13: HH × SS interaction for density.

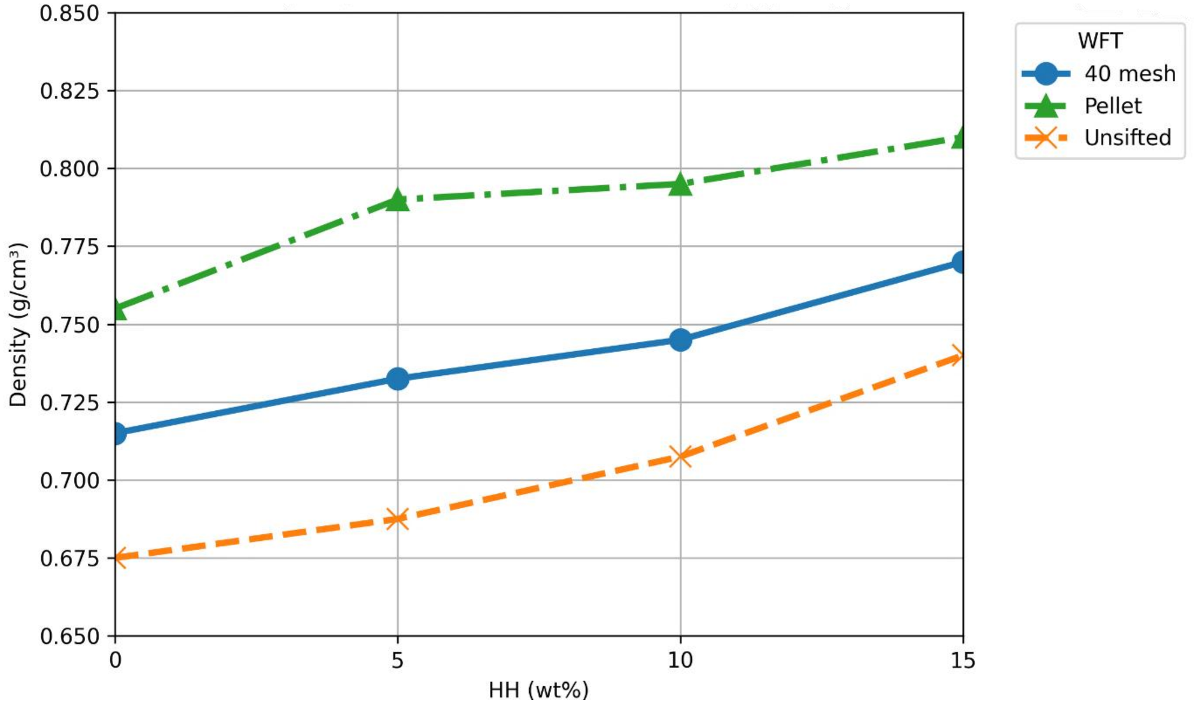

Figure S14: HH × WFT interaction for density.

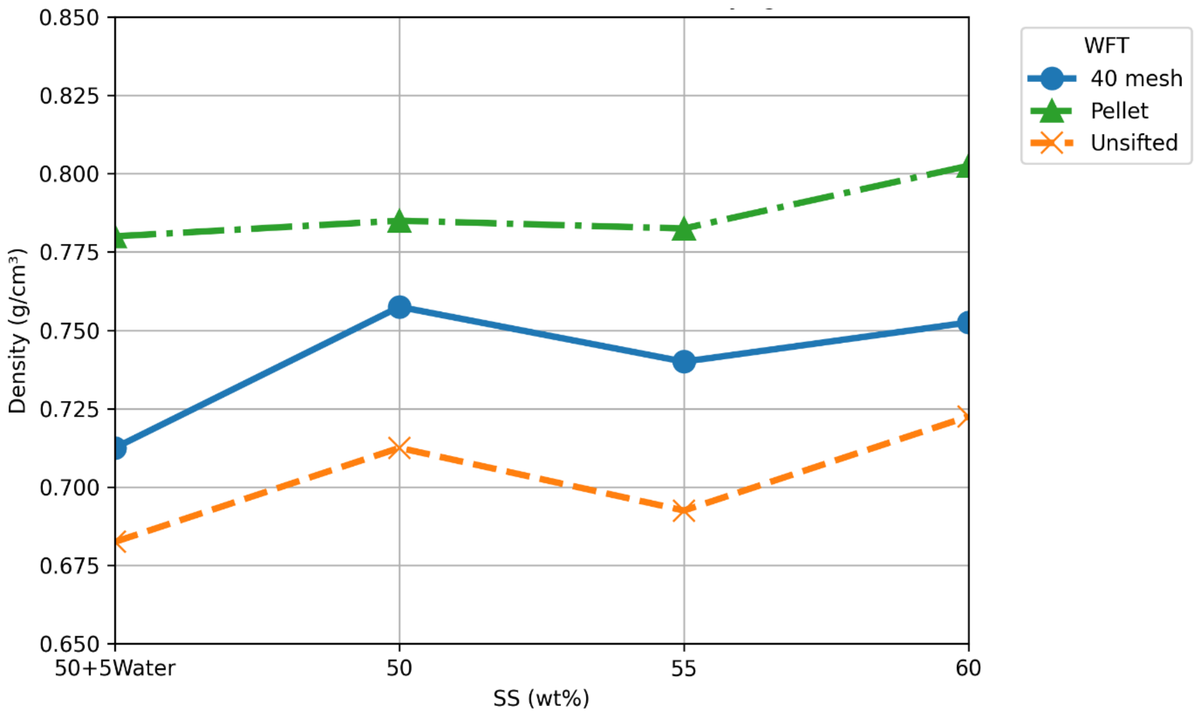

Figure S15: SS × WFT interaction for density.
